# BAP1 and PBRM1 in metastatic clear cell renal cell carcinoma: tumor heterogeneity and concordance with paired primary tumor

**DOI:** 10.1186/s12894-017-0209-3

**Published:** 2017-03-21

**Authors:** Jeanette E. Eckel-Passow, Daniel J. Serie, John C. Cheville, Thai H. Ho, Payal Kapur, James Brugarolas, R. Houston Thompson, Bradley C. Leibovich, Eugene D. Kwon, Richard W. Joseph, Alexander S. Parker

**Affiliations:** 1 Division of Biomedical Statistics and Informatics, Mayo Clinic, Rochester, MN USA; 2 Department of Health Sciences Research, Mayo Clinic, 4500 San Pablo Road, Jacksonville, FL 32224 USA; 3 Laboratory Medicine and Pathology, Mayo Clinic, Rochester, MN USA; 4 Division of Hematology and Medical Oncology, Mayo Clinic, Scottsdale, AZ USA; 5 Department of Pathology, University of Texas Southwestern Medical Center, Dallas, TX USA; 6 Kidney Cancer Program, Simmons Comprehensive Cancer Center, University of Texas Southwestern Medical Center, Dallas, TX USA; 7 Division of Hematology-Oncology, University of Texas Southwestern Medical Center, Dallas, TX USA; 8 Department of Urology, Mayo Clinic, Rochester, MN USA; 9 Division of Hematology/Oncology, Mayo Clinic, Jacksonville, FL USA

**Keywords:** Clonal, Prognostic, Immunohistochemistry

## Abstract

**Background:**

BAP1 and PBRM1 are frequently mutated in primary clear cell renal cell carcinoma (ccRCC) tumors; however, the frequency and clinical relevance of these mutations in metastatic ccRCC tumors is unknown. Additionally, while intra-tumor heterogeneity has been shown to be common in primary ccRCC, little is known regarding heterogeneity in metastatic ccRCC tumors.

**Materials and methods:**

We analyzed BAP1 and PBRM1 loss of protein expression in patient-matched primary and metastatic tumors from 97 patients. Expression was determined using a validated immunohistochemistry assay, which has been shown to be correlated with mutation status.

**Results:**

Of the 97 patients evaluated, 20 and 57% showed loss of BAP1 and PBRM1 in their primary tumors, respectively. Comparing expression across patient-matched primary-metastatic tumor pairs, 98 and 90% had concordant BAP1 and PBRM1 expression, respectively. Both patients who demonstrated discordant BAP1 expression showed loss of BAP1 expression during progression to metastatic ccRCC. Similarly, seven of the ten patients that demonstrated discordant PBRM1 expression showed loss of PBRM1 expression during progression to metastatic ccRCC. We evaluated intra-metastatic tumor heterogeneity using 12 patients who had multiple blocks available from the same tumor with representative pathology; 100 and 92% showed concordant BAP1 and PBRM1 expression, respectively. Amongst 32 patients who had serial metastatic tumors available, both BAP1 and PBRM1 had 97% concordant expression.

**Conclusions:**

We observed minimal intra- and inter- tumor heterogeneity in metastatic ccRCC tumors. Patients with discordant BAP1 or PBRM1 expression across their matched primary and metastatic tumors usually showed loss of expression during progression to metastatic ccRCC.

## Background

Mutations that cause loss of expression of BAP1 and PBRM1 are two of the most frequently occurring molecular events in primary clear cell renal cell carcinoma (ccRCC) with a prevalence of approximately 10 and 40%, respectively [1–3]. Both of these genes are located on chromosome arm 3p, which is deleted in approximately 90% of ccRCC patients. While mutations in BAP1 and PBRM1 have been shown to be associated with poor cancer-specific survival [1, 4, 5], the frequency of these mutations (and their clinical relevance) in metastatic ccRCC tumors is unknown. Related to this, Gerlinger and colleagues compared mutations from patient-matched primary and metastatic tumors in four patients; all four were BAP1 wild type and two had PBRM1 mutations [6, 7]. One of the two patients with a PBRM1 mutation (EV002) had a PBRM1 mutation in all six biopsies from the primary tumor as well as in a biopsy from a metastasis obtained at the time of disease progression on everolimus treatment. The other patient with a PBRM1 mutation (RMH004) had a mutation in three of the five biopsies from the primary tumor and a different PBRM1 mutation in a tumor thrombus from the renal vein. The value of this initial exploration notwithstanding, investigations focused on larger cohorts of patients with matched primary and metastatic ccRCC tumors are necessary to obtain estimates of the prevalence of BAP1 and PBRM1 mutations in metastatic ccRCC tumors and to better inform the potential value of these alterations as potential biomarkers for response to therapy.

When evaluating candidate tumor-based biomarkers for metastatic ccRCC, it is important to acknowledge that previous investigators have reported evidence of intra-tumor molecular heterogeneity in primary ccRCC. Specifically, authors of two studies evaluated molecular heterogeneity by performing DNA sequencing on serial biopsies taken from multiple regions of primary ccRCC tumors [6, 7]. In doing so, they observed spatial heterogeneity in every tumor evaluated and thus concluded that a single tumor biopsy gives only a small glimpse into the molecular profile of a primary ccRCC tumor. Of note, the authors in each study did not account for tumor grade and presence of necrosis in evaluating intra-tumor heterogeneity, two features that are well reported to be prognostic indicators for patients with ccRCC. Thus, their observation of intra-tumor molecular heterogeneity is not surprising given that the multiple biopsies could represent areas of the tumor with varying tumor purity as well as different aggressive pathological features. In fact, ccRCC biomarkers have been shown to be associated with pathological features of aggressiveness [8–11]. Thus, with respect to the aforementioned need to evaluate BAP1 and PBRM1 in metastatic ccRCC, these efforts should account for key underlying pathologic features of the tumor.

Motivated by gaps in the literature on BAP1 and PBRM1 in metastatic ccRCC, our objective was four-fold. First, we evaluated whether loss of expression of BAP1 and PBRM1 is a molecular homogenous or heterogeneous event within metastatic ccRCC tumors. Second, we determined the prevalence of loss of BAP1 and PBRM1 expression in metastatic ccRCC. Third, we assessed whether loss of BAP1 and PBRM1 expression in metastatic tumors is associated with cancer-specific outcome. Lastly, we evaluated the concordance of loss of BAP1 and PBRM1 expression across patient-matched primary and metastatic tumors in a large cohort of ccRCC patients.

## Methods

### Patient selection and pathology review

We identified 111 patients at Mayo Clinic Rochester who were treated surgically for ccRCC between 1990 and 2005, had synchronous (M1) or metachronous (M0 at presentation) ccRCC metastases, underwent metastasectomy for at least one of their metastatic tumors and had formalin-fixed, paraffin-embedded (FFPE) tissue available from their primary tumor and at least one metastatic tumor. For the purposes of this study, multifocal renal tumors and contralateral renal tumors were not considered as metastatic. A single pathologist (JCC) comprehensively reviewed all tumors to confirm histological subtype (1997 AJCC/UICC classification), 2010 tumor stage, 2012 ISUP tumor grade, tumor size, and presence of coagulative tumor necrosis and sarcomatoid differentiation. The FFPE block(s) that was most representative of the tumor (highest grade and presence of necrosis) was identified. If multiple blocks were identified as representative of the tumor, then all blocks were analyzed in order to evaluate intra-tumor heterogeneity across regions of the metastatic tumor that have equivalent pathological features. This study was approved by the Mayo Clinic IRB.

### BAP1 and PBRM1

Five-μm thick FFPE sections were stained using a validated and published immunohistochemical (IHC) method [2]. Blinded to the paired nature of the samples, each stained slide was reviewed to determine loss of expression of BAP1 and PBRM1. As described previously [1, 4, 5], positive staining in the background stromal cells and intratumoral lymphocytes was used as a positive internal control. Tumors were categorized as PBRM1 (BAP1) positive when tumors expressed strong diffuse nuclear staining and PBRM1 (BAP1) negative when tumor cells showed a diffuse lack of nuclear staining. In a small number of tumors only a distinct tumor nodule or area showed absent nuclear staining; these focal negative areas were thought to represent subclones of the tumor. Since some tumor cells showed lack of nuclear staining, they were deemed to be PBRM1 (BAP1) negative. Additionally, a small number of tumors showed weak nuclear staining and these weak positive cases were deemed PBRM1 (BAP1) positive. The IHC assays for BAP1 and PBRM1 have been validated and have been shown to be correlated with mutation status [2, 4].

### Statistical methods

Protein expression of PBRM1 and BAP1 were dichotomized as positive or negative, as described above. Concordance between patient-matched primary and metastatic tumors was calculated as the percentage of pairs where the primary and patient-matched metastatic tumor had the same designation. Cox proportional hazards regression was used to determine if expression in metastatic tumors was associated with ccRCC-specific survival adjusting for age at diagnosis; dichotomized expression was modeled as a time-dependent covariate. If a patient had multiple simultaneous metastases with discordant PBRM1 (BAP1) status, then the tumor with loss of expression was used in the Cox model [12]. For patients that had multiple blocks from the same metastatic tumor stained for BAP1 and PBRM1 (to estimate intra-metastatic tumor heterogeneity; each replicate block had the same tumor grade and necrosis status) we randomly chose one block to represent the metastatic tumor in the Cox model. *P*-values (*p*) <0.05 were considered statistically significant.

## Results

### Patient characteristics

Our cohort entailed 111 patients who had a primary ccRCC tumor and at least one ccRCC metastatic tumor available for molecular staining (Table 1). A total of 158 patient-matched metastases were available for these 111 patients (Table 2 and Table 3). The median time from nephrectomy to first metachronous metastasis was 1.80 years (min = 31 days, max = 10.73 years). Pulmonary metastases were the most common, accounting for 38% of all metastases (Table 2).

**Table 1:** Clinical characteristics of the primary-metastatic cohort and pathological information associated with the primary ccRCC tumor

	M0 at presentation (*N* = 57)	M1 (*N* = 54)	Total (*N* = 111)
Gender			
No	15 (26.3%)	14 (25.9%)	29 (26.1%)
Yes	42 (73.7%)	40 (74.1%)	82 (73.9%)
Age at Surgery (years)			
Mean	62.0	58.5	60.3
Median	63.6	59.1	61.4
Range	(34.9–78.8)	(38.2–73.7)	(34.9–78.8)
Max Tumor Size (cm)			
Mean	9.6	10.9	10.2
Median	9.0	10.0	9.5
Range	(2.5–18.0)	(2.1–23.0)	(2.1–23.0)
2010 pT			
Missing	0	1	1
1A	3 (5.3%)	1 (1.9%)	4 (3.6%)
1B	8 (14.0%)	8 (15.1%)	16 (14.5%)
2A	14 (24.6%)	6 (11.3%)	20 (18.2%)
2B	4 (7.0%)	5 (9.4%)	9 (8.2%)
3A	17 (29.8%)	20 (37.7%)	37 (33.6%)
3B	7 (12.3%)	4 (7.5%)	11 (10.0%)
3C	2 (3.5%)	1 (1.9%)	3 (2.7%)
4	2 (3.5%)	8 (15.1%)	10 (9.1%)
2010 pN			
0	18 (31.6%)	20 (37.0%)	38 (34.2%)
1	4 (7.0%)	9 (16.7%)	13 (11.7%)
X	35 (61.4%)	25 (46.3%)	60 (54.1%)
TNM Stage			
I	11 (19.3%)	0 (0.0%)	11 (9.9%)
II	18 (31.6%)	0 (0.0%)	18 (16.2%)
III	26 (45.6%)	0 (0.0%)	26 (23.4%)
IV	2 (3.5%)	54 (100.0%)	56 (50.5%)
Grade			
1	1 (1.8%)	1 (1.9%)	2 (1.8%)
2	14 (24.6%)	5 (9.3%)	19 (17.1%)
3	32 (56.1%)	31 (57.4%)	63 (56.8%)
4	10 (17.5%)	17 (31.5%)	27 (24.3%)
BAP1 IHC in Primary Tumor			
Negative	13 (27.7%)	6 (12.0%)	19 (19.6%)
Positive	34 (72.3%)	44 (88.0%)	78 (80.4%)
NA^a^			14
PBRM1 IHC in Primary Tumor			
Negative	28 (60.9%)	27 (52.9%)	55 (56.7%)
Positive	18 (39.1%)	24 (47.1%)	42 (43.2%)
NA^a^			14
Number of Longitudinal Metastases			
1	34 (59.6%)	41 (75.9%)	75 (67.6%)
2	15 (26.3%)	12 (22.2%)	27 (24.3%)
3	6 (10.5%)	1 (1.9%)	7 (6.3%)
4	2 (3.5%)	0 (0.0%)	2 (1.8%)

^a^Denotes that the IHC stain was unsuccessful

**Table 2:** Clinical and pathological features associated with the metastatic tumors

	M0 at presentation (*N* = 90)	M1 (*N* = 68)	Total (*N* = 158)
Year of Metastasectomy			
Median	1999	1998	1999
Range	(1991–2005)	(1990–2004)	(1990–2000)
Metastatic Site			
BONE	9 (10.0%)	10 (14.7%)	19 (12.0%)
BOWEL	1 (1.1%)	0 (0.0%)	1 (0.6%)
BRAIN	7 (7.8%)	4 (5.9%)	11 (7.0%)
CONTRALATERAL ADRENAL	3 (3.3%)	5 (7.4%)	8 (5.1%)
HEART	0 (0.0%)	1 (1.5%)	1 (0.6%)
IPSILATERAL ADRENAL	2 (2.2%)	8 (11.8%)	10 (6.3%)
LIVER	4 (4.4%)	5 (7.4%)	9 (5.7%)
MUSCLE	0 (0.0%)	1 (1.5%)	1 (0.6%)
NON-REGIONAL NODES	9 (10.0%)	1 (1.5%)	10 (6.3%)
OMENTUM	0 (0.0%)	1 (1.5%)	1 (0.6%)
OTHER	6 (6.7%)	8 (11.8%)	14 (8.9%)
PANCREAS	5 (5.6%)	2 (2.9%)	7 (4.4%)
PULMONARY	40 (44.4%)	20 (29.4%)	60 (38.0%)
SKIN	2 (2.2%)	2 (2.9%)	4 (2.5%)
SPLEEN	1 (1.1%)	0 (0.0%)	1 (0.6%)
THYROID	1 (1.1%)	0 (0.0%)	1 (0.6%)
Metastatic Grade			
2	16 (17.8%)	13 (19.1%)	29 (18.4%)
3	60 (66.7%)	36 (52.9%)	96 (60.8%)
4	14 (15.6%)	19 (27.9%)	33 (20.9%)
Metastatic Necrosis			
No	57 (63.3%)	38 (55.9%)	95 (60.1%)
Yes	33 (36.7%)	30 (44.1%)	63 (39.9%)
Metastatic Sarcomatoid			
No	86 (95.6%)	62 (91.2%)	148 (93.7%)
Yes	4 (4.4%)	6 (8.8%)	10 (6.3%)

**Table 3:** Comparison of primary tumor with a total of 158 patient-matched metastatic tumors

	Primary Tumor	Metastatic Tumor	N (%)
Grade	1	2	2 (1.3)
	1	3	1 (0.6)
	2	2	11 (7.0)
	2	3	17 (10.8)
	3	2	14 (8.9)
	3	3	64 (40.5)
	3	4	12 (7.6)
	4	2	2 (1.3)
	4	3	14 (8.9)
	4	4	21 (13.3)
Necrosis	No	No	52 (32.9)
	No	Yes	16 (10.1)
	Yes	No	43 (27.2)
	Yes	Yes	47 (29.7)
Sarcomatoid	No	No	136 (86.1)
	No	Yes	4 (2.5)
	Yes	No	12 (7.6)
	Yes	Yes	6 (3.8)

### Concordance across patient-matched primary and metastatic ccRCC tumors

#### BAP1

Of the available 111 patients, 97 (87%) primary ccRCC tumors successfully stained for BAP1 (Table 1) and 20% showed loss of BAP1 expression (IHC negative). We analyzed a total of 138 metastatic tumors from these 97 patients. Overall concordance between patient-matched primary and metastatic tumors from the 97 patients was 98%: 100% in metachronous and 96% in synchronous metastatic tumors (Table 4). With respect to the two patients with discordant primary-metastatic tumor pairs, one patient had a synchronous pulmonary metastasis and one patient had a synchronous bone metastasis. The primary tumor for both patients was IHC positive and the metastatic tumors were IHC negative.

**Table 4:** Percent (%) concordance across primary-metastatic tumor pairs for 97 patients

	% Concordant	
	PBRM1	BAP1
Overall	89.7	97.9
M0 at presentation	89.1	100
M1	90.2	96.0
Pulmonary	85.3	97.1
M0 at presentation	82.4	100
M1	88.2	94.1
Non-pulmonary	92.1	98.4
M0 at presentation	93.1	100
M1	91.2	97.0

#### PBRM1

Ninety-seven (87%) primary tumors successfully stained for PBRM1 (Table 1) and 57% showed loss of PBRM1 (IHC negative). Ninety-six of these 97 patients also had BAP1 status available: 6 (6%) had loss of both BAP1 and PBRM1, 49 (51%) had loss of PBRM1 only, 13 (14%) had loss of BAP1 only and 28 (29%) lost neither BAP1 nor PBRM1. For the 97 patients with staining of PBRM1 in the primary tumor, we analyzed a total of 138 patient-matched metastatic tumors. Overall concordance between patient-matched primary and metastatic tumors from the 97 patients was 90%: 89% in metachronous and 90% in synchronous metastatic tumors (Table 4). Discordance was observed across metastatic sites. Of the 10 patients that had at least one discordant primary-metastatic tumor pair, five had synchronous metastatic tumors and five had metachronous metastatic tumors. Among these 10 patients, seven (70%) demonstrated loss of PBRM1 during progression to metastatic ccRCC.

### Intra-metastatic tumor heterogeneity

Replicate blocks were analyzed for BAP1 and PBRM1 expression in 12 metastatic tumors in order to investigate intra-tumor heterogeneity (median = 2, max = 5 replicate blocks per metastatic tumor). Of these, we observed 100% concordance in BAP1 status and 92% concordance in PBRM1 status.

### Concordance across longitudinal metastatic ccRCC tumors

BAP1 and PBRM1 staining was performed on longitudinal metastatic tumors for 32 patients (median = 2, max = 4 metastatic tumors per patient). We observed inter-metastatic tumor heterogeneity of BAP1 in one (3%) patient. The primary tumor for this patient was BAP1 IHC positive, the first bone metastasis (synchronous) was IHC negative and the second bone metastasis (diagnosed approximately 9 months later) was IHC positive. Similarly, we observed inter-metastatic tumor heterogeneity of PBRM1 in one (3%) patient. This patient had two available synchronous pulmonary metastases (i.e., at the time of surgery, these two metastases were determined to be different pulmonary nodules): the primary tumor was PBRM1 IHC positive, one pulmonary nodule was IHC negative and the other pulmonary nodule was IHC positive.

### Associations of metastatic tumor expression with RCC-specific survival

We did not observe a statistically significantly association with ccRCC-specific survival for either metastatic expression of BAP1 (HR = 1.29, 95% confidence interval (CI): 0.76–2.19, *p* = 0.34) or PBRM1 (HR = 0.92, 95% CI: 0.55–1.52, *p* = 0.79).

## Discussion

Chromosome arm 3p loss is a common event in primary ccRCC tumors and four of the most commonly mutated genes are all located on 3p: VHL, BAP1, PBRM1 and SETD2. We evaluated BAP1 and PBRM1 loss of protein expression in a large cohort of patient-matched primary and metastatic ccRCC tumors. Additionally, we examined molecular heterogeneity in metastatic ccRCC tumors.

While previous investigators have reported evidence of intra-tumor molecular heterogeneity in primary ccRCC [6, 7], little is known regarding heterogeneity in metastatic ccRCC tumors. The current research evaluating intra-tumor heterogeneity in primary ccRCC tumors has been performed comparing multiple biopsy samples derived from the same tumor. Such analyses provide important information to determine if biopsy samples can be used for selecting biomarker-based neoadjuvant systemic therapy. Our objective herein was to determine if intra-tumor molecular heterogeneity exists across multiple samples from the same surgically-resected tumor that all have similar pathological feature. It is well known that the drivers, in terms of behavior based on conventional morphology, are tumor grade and necrosis and the areas of a tumor with the highest grade and presence of necrosis are likely enriched for the molecular drivers of metastasis. In fact, for studies evaluating candidate tumor biomarkers, it has been suggested that areas of high nuclear grade should be analyzed [12]. Indeed, the concordance in morphology between patient-matched primary and metastatic tumors supports this recommendation [13]. Thus, we evaluated if intra-tumor heterogeneity exists across samples from the same tumor with similar pathological features. Similar analyses have been performed comparing ccA/ccB ccRCC gene expression subtypes for this cohort of patient-matched primary and metastatic tumors [14]. Herein, we observed minimal intra- and inter-tumor heterogeneity of BAP1 and PBRM1 loss of expression in metastatic ccRCC tumors. Together, these results suggest that multiple samples from the same metastatic tumor may not be necessary if stringent pathological review is incorporated. However, similar analyses should be performed on additional molecular markers to verify that the results are concordant for other markers.

Previous investigators have reported that BAP1 mutations occur in approximately 10% of ccRCC tumors [1, 2, 4] and loss of BAP1 expression is associated with reduced survival [1]. The higher prevalence of loss of BAP1 expression observed herein is likely due to our high-risk (metastatic) cohort. We observed that loss of BAP1 protein expression was nearly 100% concordant between patient-matched primary and metastatic tumors. This further underscores that BAP1 is likely a truncal mutation and essential to both ccRCC development and pathogenesis [7, 15]. The two patients that were discordant demonstrated loss of BAP1 during progression to metastatic ccRCC, which suggests the presence of a clone in the primary tumor that had lost BAP1. While loss of BAP1 expression in primary ccRCC tumors has been shown to be prognostic [1, 16], we did not observe a statistically significant association in metastatic ccRCC tumors. However, due to the low prevalence of loss of BAP1 expression, associations with outcome should be examined in larger cohorts of metastatic ccRCC tumors.

PBRM1 mutations have been shown to be prevalent in approximately 40% of ccRCC tumors [1, 3, 4] and loss of PBRM1 expression has been shown to be associated with overall survival [4]. The higher prevalence of loss of PBRM1 expression observed herein is likely due to the cohort of high-risk patients. We observed a negative genetic interaction between BAP1 and PBRM1, confirming a result that was reported previously [5]. Under independence we would have expected 11% of the primary tumors to show loss of both BAP1 and PBRM1; however, 6% of our primary tumors had loss of expression for both genes. We observed high concordance of loss of PBRM1 expression between patient-matched primary and metastatic tumors. Of the 10 patients that demonstrated discordance, 70% had primary tumors that were IHC positive and subsequently the metastatic tumors demonstrated PBRM1 loss of expression. This corroborates our findings that when patient-matched primary and metastatic tumors had discordant ccA/ccB molecular subtype, 80% of these discordant patients progressed from having a primary tumor classified as ccA and a metastatic tumor classified as ccB [14]. Furthermore, when evaluating pathological characteristics across patient-matched primary and metastatic RCC tumors, trends for higher grade and tumor necrosis in the metastatic tumor have been observed [13]. Overall, our results suggest that there may be a clone in the primary tumor that had lost PBRM1. Discordance was observed across multiple metastatic sites, and thus, does not appear to be site specific. Additionally, we did not observe an association between metastatic PBRM1 expression and ccRCC-specific survival.

We acknowledge that there may be systematic differences between subjects who underwent metastasectomy and subjects with distant metastases that were not treated surgically. However, tissue will only be available from patients undergoing metastasectomy, so this limitation is unavoidable. We used IHC to measure BAP1 and PBRM1 protein loss instead of directly determining BAP1 and PBRM1 mutation status. Thus, even though the IHC assays for BAP1 and PBRM1 have been validated and shown to be correlated with mutation status in previous studies [2, 4], the impact of mutations in these proteins could not be determined. IHC is both more cost effective and easily applied to formalin-fixed paraffin-embedded specimens, which are both important considerations when attempting to analyze hundreds of clinically-stored tissue specimens. We only analyzed metastatic tumors from patients whom we also had a patient-matched surgically-resected primary ccRCC tumor available for analysis. Because we ignored metastatic tumors from patients who did not have a nephrectomy at our institution, we may have a biased sample of metastatic tumors. Thus, future studies examining the molecular characteristics of metastatic tumors and identification of prognostic markers should evaluate all available metastatic tumors.

## Conclusions

We observed minimal molecular heterogeneity across sections from the same metastatic tumor that had similar pathological features. With respect to progression from primary-to-metastatic ccRCC, loss of BAP1 and PBRM1 expression demonstrated minimal heterogeneity. Thus, BAP1 and PBRM1 are likely key events in both ccRCC development and progression to metastasis. Currently, the presence or absence of either of these mutations does not guide clinical decision making, especially in regards to choosing therapy for metastatic disease. However, there are multiple potential therapeutic targets where these mutations could be clinically relevant in the future. And, given the high concordance of these mutations between primary and metastatic tumors, testing the more readily-available primary tumor might be sufficient. Going forward, analyzing serial samples from the same patient and mapping the clonal evolution of ccRCC will aid in understanding the molecular alterations that underlie ccRCC pathogenesis [6, 7, 17–22]. As demonstrated herein, this approach needs to be taken using larger cohorts and associations with pathological features and outcome should be conducted.

## Abbreviations

ccRCC: clear cell renal cell carcinoma
CI: Confidence interval
FFPE: Formalin-fixed, paraffin-embedded
HR: Hazard ratio
IHC: Immunohistochemical
P: *p*-value
RCC: Renal cell carcinoma
